# *Candida albicans* Modulates Murine and Human Beta Defensin-1 during Vaginitis

**DOI:** 10.3390/jof8010020

**Published:** 2021-12-28

**Authors:** María Soledad Miró, Juan Pablo Caeiro, Emilse Rodriguez, Lara Vargas, Cecilia Vigezzi, Paula A. Icely, Graciela D. V. Castillo, Ana I. Azcurra, Claudio D. Abiega, Fernando O. Riera, Claudia E. Sotomayor

**Affiliations:** 1 Laboratory of Innate Immunity to Fungal Pathogens, Department of Clinical Biochemistry, Faculty of Chemical Sciences, National University of Córdoba, Córdoba 5000, Argentina; msmiro@unc.edu.ar (M.S.M.); erodriguez@unc.edu.ar (E.R.); cvigezzi@fcq.unc.edu.ar (C.V.); picely@fcq.uc.edu.ar (P.A.I.); 2 Center for Research in Clinical Biochemistry and Immunology, CIBICI-CONICET, Córdoba 5000, Argentina; 3 Infectious Disease Section, Hospital Privado Universitario de Córdoba, Córdoba 5000, Argentina; jpcaeiro3@gmail.com (J.P.C.); cabiega@gmail.com (C.D.A.); 4 Infectology Department, Sanatorio Allende, Córdoba 5000, Argentina; lvargas@gmail.com (L.V.); friera@hotmail.com (F.O.R.); 5 Department of Buccal Biology, Faculty of Odontology, National University of Córdoba, Córdoba 5000, Argentina; graciela.castillo@unc.edu.ar (G.D.V.C.); ana.azcurra@unc.edu.ar (A.I.A.)

**Keywords:** vaginitis, vulvovaginal candidiasis, beta-defensin, antimicrobial peptides, mBD1, hBD1, fungal immunity, cytokines, *Candida albicans*

## Abstract

Vulvovaginal candidiasis (VVC) and recurrent vulvovaginal candidiasis (RVVC) are two forms of a disease caused by *Candida* spp. β-defensin (BD) is one of the most important families of antimicrobial peptides in the female genital tract and includes molecules that exert essential local functions as antimicrobial and PMN chemoattractant peptides. However, the information on their role during murine and human VVC and RVVC is limited. Thus, we analyzed the behavior and contribution of BD1 to the local response in a VVC mice model and the local cytokine profile and human BD1 and BD3 expression in cervicovaginal lavage from patients with VVC and RVVC. We demonstrated that, in patients with RVVC BD1, mRNA and protein expression were severely diminished and that the aspartate proteinase and lipase secreted by *C. albicans* are involved in that decrease. This study provides novel information about the pathogenesis of VVC and describes a highly efficient *C. albicans* escape strategy for perpetuating the infection; these results may contribute to the development of new or combined treatment approaches.

## 1. Introduction 

Vulvovaginal candidiasis (VVC) is a widespread disease caused by *Candida* species that affects a significant number of women of reproductive age. Up to 75% of women experience acute VVC (AVVC) at least once in their lifetime, which is associated with risk factors such as pregnancy, oral contraceptives, diabetes mellitus, long-term broad-spectrum antibiotic treatment, and steroid and immunosuppressive therapies [1]. Moreover, up to 9% of women experience recurrent vulvovaginal candidiasis (RVVC), a more distressing condition characterized by at least four episodes of AVVC per year [2]; however, the great majority of patients with RVVC do not display any of the associated risk factors. In their systemic review, Denning et al. reported that RVVC affects more than 130 million women in any given year, with a global annual prevalence of 3871 cases per 100,000 individuals, highlighting the significant global burden that this infection represents [3,4].

*Candida* species coexist with bacterial communities of the vaginal microbiome in a harmonious balance and under the strict control of the local immune response. The disruption of this balance caused, for example, by dysbiosis or an abnormal host mucosal immune response to *Candida* favors fungal overgrowth, morphogenetic changes, expression of hyphae-associated virulence factors, and tissue invasion. In the complex scenario of pathogen–host interactions, fungal factors and host response mechanisms elicit inflammatory signals that promote the recruitment of polymorphonuclear neutrophils (PMNs) to the lamina propria and the vaginal lumen and the release of innate immune mediators [5,6,7]. This strong and deregulated proinflammatory response against *Candida* is associated with vulvovaginal symptoms such as vaginal discharge, itching, burning, and dyspareunia, which can vary from moderate to severe [1,6,8]. Therefore, RVVC, which is associated with ineffective treatments, will invariably affect a person’s life quality.

The vaginal epithelium plays a vital role in the innate immunity of the genital tract by providing a physical barrier and producing immune mediators and antimicrobial peptides (AMPs) [8]. AMPs are evolutionarily conserved effector molecules that exhibit a broad spectrum of antimicrobial activity against bacteria, fungi, and viruses [9]. β-defensins (BDs), one of the most important families of AMPs, are small (4–6 kDa) cationic peptides that present antimicrobial properties and can also exert chemotactic activity by recruiting PMN, immature dendritic cells, and memory T cells to the site of infection by binding to the chemokine receptor CCR6 [9,10]. Human BD1 (hBD1) is constitutively expressed in uninflamed normal mucosa, and it is considered relevant in the female reproductive tract (endometrium, vagina, cervix, and fallopian tubes) [11] as well as the most critical AMP in epithelial defense against pathogens [12]. Different single nucleotide polymorphisms within the hBD1 encoding gene can affect its expression, causing individuals to be more susceptible to infection [12,13]. It has been reported that recombinant mBD1, a murine homolog of hBD1 [14], can display direct fungicidal activity [15]. BD regulation has been well documented in *Candida* infection, especially in the gastrointestinal tract, and also in the course of human esophagitis [16], oropharyngeal and gastric murine models [17,18], in vitro studies [17,19], and in human oral infections with reduced levels of salivary hDB1-3 [20]. In a murine model of *C. albicans* oral infection, Tomalka et al. [21] reported that, at early times postinfection (D3 pi), mBD1 deficient mice exhibit 10-fold higher CFU than WT animals and lower PMNs infiltration in the mucosa. Further, it has been described that this deficiency also impacts the production of IL-1β, IL-6, and IL-17. Nonetheless, the available evidence on *C. albicans* persistence, the mechanisms involved in the inflammatory response, and the role of BDs in the pathogenesis of human AVVC and RVVC is limited. Therefore, in the present work, we evaluated the role of BD1 in AVVC using murine AVVC models and by analyzing human samples obtained from patients with AVVC or RVVC. Our results demonstrated that, although *C. albicans* presence upregulates the expression of constitutive BD1 (murine and human) during the acute invasion event, when the host–fungus interaction occurs in a permissive host, *C. albicans* and its secreted virulence factors strongly decrease BD1 levels, favoring the establishment of the fungus and perpetuation of the infection.

## 2. Materials and Methods

### 2.1. Ethics Statement

This work was approved by the Institutional Review Board (IRB) of Sanatorio Allende (Sanatorium Allende, Cordoba, Argentina) and Hospital Privado Universitario de Córdoba (Private University Hospital of Córdoba) and by the Provincial Registries of Health Research (RePIS), Cordoba, Argentina (approval number 4-168). All the patients enrolled in the study provided written informed consent prior to participation, in accordance with the Declaration of Helsinki. Animals were housed at the Animal Care Facility of the Center for Research in Clinical Biochemistry and Immunology, maintained under a standard light cycle (12 h light/dark), and allowed free access to water and food. Animal experimental protocols were approved by the Institutional Animal Care and Use Committee (IACUC) of the Faculty of Chemical Sciences, National University of Córdoba (approval number EUNC0045378/RD939). All efforts were made to minimize animals’ suffering during each experiment.

### 2.2. Candida spp. and Determination of Hydrolytic Enzymes Activity

*C. albicans* ATCC-36801 [22,23], SC5314 [24], and the clinical isolate *FKS1-*R1361R/H [25] were stored in glycerol at −80 °C. The clinical isolates recovered from patients with AVVC and RVVC were identified by Maldi-Tof (Biomerieux, Craponne, France), and their virulence profiles were evaluated [24,26]. One strain isolated from RVVC patients, with an established virulence profile, was used for in vivo and in vitro studies and was identified as *C. albicans* RVVC. The colonies were grown on SDA-media (Britania, Los Patos, Argentina) at room temperature (RT). For each infection, yeast cells were harvested after 48 h of culture, centrifuged at 1000× *g*, washed twice in sterile PBS, and counted in a Neubauer hemocytometer chamber. The inoculum used in each experiment was standardized at 2.5 × 10^8^ yeast/mL in PBS. The number of viable cells was assessed by triplicate on SDA-media after 48 h of incubation at RT.

In order to semiquantify acid aspartic protease (SAP) activity, the agar method supplemented with BSA was used (dextrose 2%, KH_2_PO_4_ 0.1%, MgSO_4_ 0.05%, agar 2% mixed after cooling to 50 °C with 1% BSA solution). *Candida* spp. suspensions (100 μL, 1 × 10^7^ cells/mL in sterile PBS) were inoculated in triplicate in wells made in the agar. The plates were incubated at 37 °C in aerobiosis for 72–96 h. Enzymatic activity was observed as a proteolysis halo around the colony, and it was calculated as the ratio between the diameters of the proteolysis halo and the colony (*Pz*SAP) [24].

Rhodamine-B plaque assay was used to identify and semiquantify lipolytic activity (LIP). To induce activity, yeasts (1 × 10^8^ *Candida* cells, after 48 h of growing in SGA) were suspended in 2 mL of 0.7% of Sabouraud broth (Britania, Buenos Aires, Argentina), 0.2 mL 10% CFS, and 0.05 mL 2.5% Tween 80 (Biopack, Buenos Aires, Argentina) and incubated for 72 h at 37 °C. Rhodamine B agar plates (1.5% agar Britania, Buenos Aires, Argentina; 1% olive oil; 0.35% Sabouraud broth; Rhodamine B 0.001%, LTD, London, UK) were inoculated with 150 μL of each suspension in triplicate wells and incubated at 37 °C in aerobiosis. After 72–96 h, the orange fluorescence diameters of the lipase diffusion halos were exposed to UV irradiation using a transilluminator (BioDoc-IT Systems, UV, Cambridge, UK). The well and the fluorescence diameters were measured to calculate the lipolytic activity (*Pz*LIP = fluorescence diameter/well diameter). We used 150 μL of Sabouraud broth as a negative control [26].

### 2.3. VVC Model

Female C57BL/6J mice (8–10 weeks old) were treated subcutaneously with 0.2 mg of β-estradiol 17-valerate (Merck-Millipore, Darmstatd, Germany) in 100 μL of sesame oil (Merck-Millipore) on days (D) 6, 3, 2, and 4 postinfection (pi). On D0, mice were inoculated intravaginally (i.v.) with 20 μL of *C. albicans* suspension (infected group; 5 × 10^6^ yeasts/PBS; *n* = 8) or PBS (uninfected group; *n* = 8). Finally, a third group was not infected nor treated (unestrogenized group; *n* = 7). *C. albicans* ATCC-36801 and *C. albicans* RVVC were used for the in vivo infection. *C. albicans* RVVC is more virulent than the ATTC-36801 strain, based on SAP activity, adherence, and biofilm-forming capacity [24,26]. Uninfected animals were used as negative controls [23].

The time course of the infection was monitored in individual mice by culturing 100 μL of serially diluted (1:10) vaginal lavages on SDA. Vaginal lavages were conducted using 70 μL of sterile PBS with repeated aspiration and agitation. Colony-forming units (CFUs) were counted after 48 h of incubation at RT and expressed as CFU/mL of vaginal lavage. Quantitative counts of CFU in lavage fluids were evaluated [22,23].

### 2.4. Immunohistochemical Analysis

The vaginas were obtained, sectioned into 3–4 μm slices, and incubated overnight at 4 °C with a rabbit antimouse β-Defensin 1 primary antibody (Santa Cruz Biotechnology, Dallas, TX, USA). Then, the sections were incubated with an antirabbit biotin-labeled antibody (Vector Labs, Burlingame, CA, USA) in PBS with 1% BSA [23,27] and finally with the VECTASTAIN^®^ Elite ABC-HRP Kit, Peroxidase (Vector Labs, Burlingame, CA, USA). Diaminobenzidine (Sigma–Aldrich, St. Louis, MO, USA) was used as chromogen substrate. The histological sections were observed with a NIKON ECLIPSE microscope (Nikon, Tokyo, Japan).

### 2.5. Flow Cytometry

Vaginal lavage (VL) cells were collected from 3–4 mice per group, resuspended in PBS-FCS 2%, and permeabilized with Cytofix/Cytoperm (BD Biosciences, East Rutherford, NJ, USA). The VL cells were then incubated with a rabbit antimouse BD1 antibody (Santa Cruz Biotechnology, Dallas, TX, USA) and finally with a secondary goat antirabbit AlexaFluor 647 antibody (Abcam, Waltham, MA, USA). All the staining steps were performed at 4 °C for 30 min. In another set of experiments, Gr-1+ and mBD1+ expression in VL cells were analyzed. First, VL cells were stained with a goat antimouse Gr-1PE conjugated antibody (BD Bioscience) and subsequently fixed and permeabilized with Cytofix/Cytoperm. Then the cells were incubated with a rabbit antimouse BD1 antibody (Santa Cruz Biotechnology, Dallas, TX, USA) and finally with a secondary goat antirabbit AlexaFluor 647 antibody. The controls used were unstained cells (negative control) and isotype control (Sigma–Aldrich, St. Louis, MO, USA). Analysis was performed on living cells by excluding dead cells by FSC vs. SSC and Singlets gating, as shown in the gating strategy Figure S1. The absolute number of mBD1+ cells in VL was calculated based on the percentage of mBD1+ cells and the total cell count of VL cells obtained using a Neubauer chamber. For the evaluation the expression of hBD1 in HeLa cells, rabbit anti-human BD1 antibody (Santa Cruz Biotechnology) and goat anti-rabbit AlexaFluor 647 secondary antibody (Abcam) were used. The assays were performed as described above. Stained cells were acquired (300,000 events) using a FACSCanto II cytometer (BD Biosciences), and the data were analyzed using the FlowJo software (Tree Star, Inc. Becton, Dickinson and Company, Ashland, OR, USA).

### 2.6. Study Participants

The patients included in this study were attending to the Hospital Privado Universitario de Córdoba and Sanatorio Allende, in Córdoba, Argentina. A total of 105 women were enrolled, and they were categorized into three groups: RVVC, presented with four episodes of VVC per year (*n* = 59); AVVC, presented with ongoing vaginal *Candida* infection and no history of previous episodes (*n* = 20); and Control, healthy individuals, were negative for *Candida* infection, and matched in age with both groups of patients (*n* = 26). All the individuals were 18–52 years old, nondiabetic, negative for HIV, sexually active, and nonpregnant. Patients with vaginal infection other than *Candida*, patients with any coinfection, menopausal women, and patients using or receiving antimicrobial therapy within the previous two weeks were excluded from the study.

Cervicovaginal lavage (CVL) was collected by washing the cervix and vagina with 3 mL of sterile saline. The samples were centrifuged at 2000 rpm, and the cells were spun down onto slides to be used in immunofluorescence assays or preserved in TRIzol Reagent (Thermo Fisher, Waltham, MA, USA) RNA extraction. Supernatants were aliquoted and stored at −80 °C.

### 2.7. Cytokine Immunoassays

Cytokines in the CVL were quantified by ELISA assay using TNF-α, IL-6, IL-1β, TGF-β, IL-22, IL-23, and IL-17 detection kits (R&D System, McKinley Place, MN, USA) [19]. The samples were previously concentrated with a Vivaspin-6 centrifugal concentrator (GE Healthcare, Chicago, IL, USA). In the murine AVVC model, the level of mouse IL-1 β in the vaginal lavage was quantified using ELISA tests (R&D System, McKinley Place, MN, USA). Absorbance was measured using a BIO-RAD microplate reader, and the concentrations were extrapolated from standard curves. The data were expressed as picogram/milliliter (pg/mL).

### 2.8. Immunofluorescence Analysis

CVL cells attached to coverslides were fixed with 4% paraformaldehyde (Sigma–Aldrich) for 40 min at RT, washed with PBS, blocked, and permeabilized with PBS containing 10% BSA (Sigma–Aldrich) 0.3% Triton X-100 (Biopack, Buenos Aires, Argentina) for 1 h at RT. Then, the CVL cells were incubated overnight at 4 °C with rabbit antihuman BD1 or rabbit antihuman BD3 antibodies (Santa Cruz Biotechnology) and next with goat antirabbit AlexaFluor 647 secondary antibody (BD Biosciences) for 30 min at RT. After DAPI counterstaining, the cells were mounted with FluorSave Reagent (Merck-Millipore), and immunofluorescence was evaluated using a LEICA-DMi8 microscope (Leica Microsystems, Wetzlar, Germany). hBD1 and hBD3 semiquantification was performed using the ImageJ software. Corrected total fluorescence was calculated as [Integrated Density—(Epithelial cell area × Mean background fluorescence)]. Mean background fluorescence was calculated using three random areas of each image that were considered negative.

### 2.9. Real-Time PCR

Real-time PCR was used to evaluate the levels of hBD mRNA in CVL recovered cells [24,28,29] using the StepOne System (Life Technologies, San Francisco, CA, USA) and SYBR Select Master Mix (Life Technologies). RNA was obtained using TRIzol (Thermo Fisher) following the manufacturer’s instructions. DNA contamination was removed with a DNA removal kit (Thermo Fisher), and cDNA was obtained using the following reagents: Reverse Transcriptase M-MLV (Promega, Madison, WI, USA), Oligo dT (Promega), dNTPs (Invitrogen, Waltham, MA, USA), and RNAsin (Promega). The program used for cDNA obtention was: 5 min at 65 °C, 1 h at 42 °C, and 10 min at 70 °C. hBD mRNA expression was evaluated with the following primers: hBD1 Fw: 5′-ATGGCCTCAGGTGGTAACTTTC, Rev: 5′-TCGGGCAGGCAGAATAGAGA; hBD2 Fw: 5′-CCTGGAACAAAATGCTGCAA, Rev: 5′-ACATGTCGCACGTCTCTGATG; hBD3 Fw: 5′-GTGAAGCCTAGCAGCTATGAGGAT, Rev: 5′-TGATTCCTCCATGACCTGGAA; and β-actin Fw: 5′-CTGGAACGGTGAAGGTGACA, Rev: 5′-TGCGTTGTTACAGGAAGTCCCTT, as housekeeping gene. The PCR program used was: 15 s at 95 °C, 30 s at 60 °C, and 40 cycles of 30 s at 72 °C. For normalization, we obtained the Ct for the gene of interest and the β-actin gene for each patient. The expression of the gene of interest relative to β-actin was expressed as 2^−ΔΔCT^. The control patients were used as the reference group.

### 2.10. Human Cell Culture

HeLa cells were cultivated at 37 °C and 5% CO_2_ in DMEM (Gibco, Waltham, MA, USA) supplemented with 10% FCS (Natocor, Cordoba, Argentina), 1% L-glutamine (Gibco), and 1% penicillin–streptomycin (Gibco). A total of 2.5 × 10^5^ cells/well were incubated in a 24-well plate (CellStart, Greiner one, Frickenhausen, Germany). After 24 h, the cells were incubated for 4 h with different *C. albicans* strains at a 1:1 or 5:1 *Candida*: cell ratio or with the following stimuli: LPS (Sigma–Aldrich) 1 μg/mL or 10 μg/mL, Pam3CSK4 100 ng/mL (Invivogen, San Diego, CA, USA), Zymosan 20 μg/mL (Sigma–Aldrich, St Louis, MO, USA), IL-1β 10 ng/mL (R&D Systems, McKinley Place, Shorewood, MN, USA), and Heat-Killed Candida (HKC) at a 1:1 ratio. After the 4 h simulation/infection of HeLa cells, the supernatants were collected, and cold PBS was added to the monolayer for 15 min in order to detach the cells. Different strategies were used to study hBD1 regulation, including a transwell culture system (Costar, Cambridge, MA USA). *C. albicans* was placed in the upper compartment of the transwell and was not in contact with the HeLa cells. Another strategy used was the addition of 625 μM Acetylsalicylic Acid (ASA; Sigma–Aldrich), a lipase inhibitor, and 1 μg/mL Pepstatin A (Sigma–Aldrich), a SAP inhibitor.

### 2.11. Statistical Analysis

Data were expressed as means ± SEM. Prior the statistical analysis, the test of data normality (Shapiro–Wilk test) was performed. Differences between groups means or ratios were assessed using one-way or two-way ANOVA followed by the Bonferroni test for multiple comparisons. A *p*-value < 0.05 was considered statistically significant. GraphPad Prism version 6.0 for Windows (GraphPad Software, San Diego, CA, USA) was used to perform the statistical analysis. All experiments were repeated at least twice to test the reproducibility of the results.

## 3. Results

### 3.1. mBD1 Expression Is Upregulated in the Vaginal Tract During VVC

**Figure 1:**
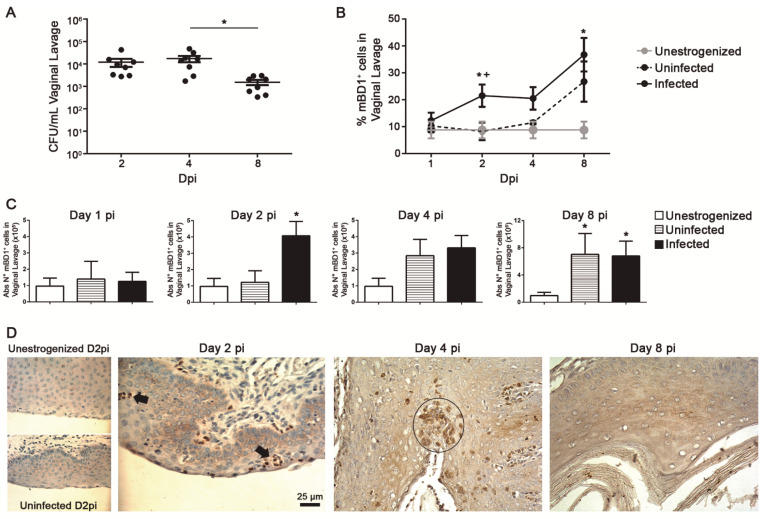
Upregulated expression of mBD1 in vaginal tract during VVC. (**A**) Fungal growth (CFU ± SEM) at D2, 4, and 8 pi in vaginal lavage of C57BL/6 mice infected i.v. with 5 × 10^6^ cells of *C. albicans* ATCC 36801. Each point represents an individual mouse (*n* = 8). Data are representative of three independent experiments. * *p* < 0.05. (**B**) Percentage of mBD1 total positive cells in vaginal lavage from C57BL/6 mice unestrogenized (*n* = 7), uninfected (*n* = 8), or infected (*n* = 8) i.v. with 5 × 10^6^ cells of *C. albicans* ATCC 36801 at different Dpi. Mean ± SEM of two independent experiments. * *p* < 0.05 infected vs. unestrogenized group; + *p* < 0.05 infected vs. uninfected group. (**C**) Absolute number of mBD1 total positive cells in vaginal lavage from C57BL/6 mice unestrogenized, uninfected, or infected i.v. with 5 × 10^6^ cells of *C. albicans* ATCC 36801 at different Dpi. Mean ± SEM of two independent experiments. * *p* < 0.05 vs. unestrogenized group. ANOVA Test. (**D**) Vaginal tissue sections from C57BL/6 mice unestrogenized (D2), uninfected (D2), or infected i.v. with 5 × 10^6^ cells of *C. albicans* ATCC 36801 at D2, 4, and 8 pi, stained with anti-mBD1 antibody. Positive cells stained brown with diaminobenzidine against the blue hematoxylin counterstaining. Images are shown at ×400 magnification. The black arrow indicates infiltrating PMNs cells, and the circle indicates the presence of microabscesses.

In order to characterize the kinetic expression profile of mBD1 during VVC, we evaluated the pattern of expression of this peptide using a well-characterized murine model of acute VVC (AVVC) in which C57BL/6 mice were i.v. infected with *C. albicans* [23]. Briefly, a group of mice was inoculated with *C. albicans* ATCC-36801 (infected group) and treated with estrogen, another group received PBS and was treated with estrogen (uninfected/estrogenized group), and the third group was neither treated nor infected (unestrogenized group). At D2 and 4 pi, the fungal burden showed similar colonization, while a decrease was observed at the end of the study (Figure 1A). At D2 pi, a significant increase in the percentage and absolute number of mBD1+ cells in VL was found in infected animals when compared to the other two groups (Figure 1B,C). At D4 pi, the percentage of mBD1+ cells was similar to that of D2 pi, and no significant differences were observed when compared to the uninfected and unestrogenized animals. The absolute number and percentage of mBD1+ cells increased in the infected and uninfected groups at D8 pi, showing that the estrogen treatment was able to modulate mBD1 expression in the vaginal tract. In the intact mucosa of both negative control groups, a basal and homogeneous distribution of mBD1 expression was observed in the stratified epithelium, with a slight increase after exposure to estrogen treatment (Figure 1D, left panels). In *C. albicans*-infected mice, progressive immunostaining in epithelial cells (EC) was observed. The PMNs infiltrate and corneal microabscesses showed a strong mBD1 positive reaction. These results indicate that the expression of this constitutive peptide is upregulated in the vaginal tract during *C. albicans* infection.

### 3.2. mBD1 Expression Is Differentially Regulated after Infection with a C. albicans Strain Recovered from an RVVC Patient

**Figure 2:**
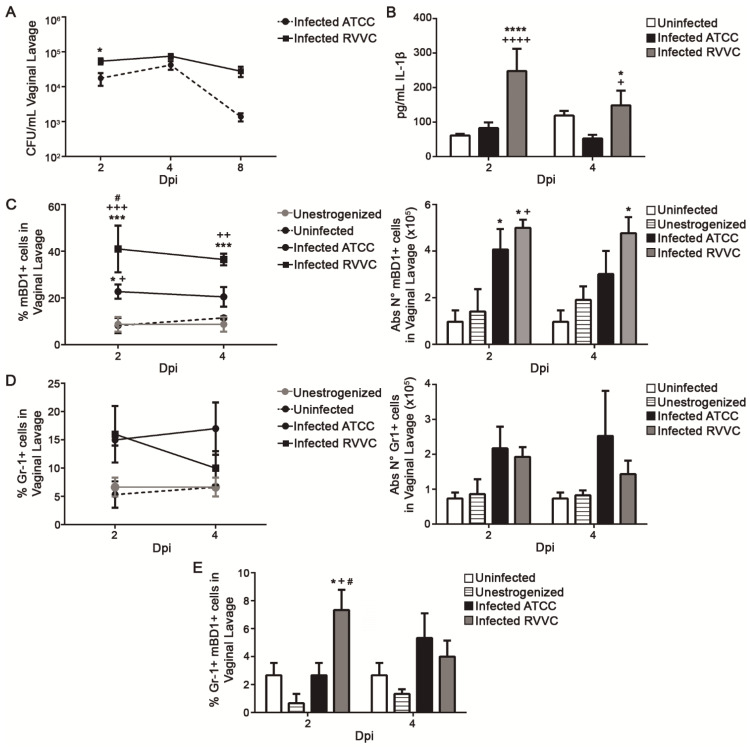
Differential expression of mouse Beta Defensin-1 (mBD1) during AVVC induced with *C. albicans* clinical or collection strains. (**A**) Fungal growth (CFU ± SEM) at days 2, 4, and 8 pi in vaginal lavage of C57BL/6 mice infected i.v. with 5 × 10^6^ cells of *C. albicans* ATCC 36801 or *C. albicans* RVVC. Mean ± SEM of three independent experiments (*n* = 8 per group). * *p* < 0.05. (**B**) IL-1β levels in vaginal lavage from uninfected and *C. albicans* ATCC- or *C. albicans* RVVC-infected animals from days 2 and 4 pi. IL-β was not detected in unestrogenized animals. Mean ± SEM of three independent experiments (*n* = 8 per group). * *p* < 0.05, **** *p* < 0.0001 infected vs. uninfected; + *p* < 0.01, ++++ *p* < 0.0001 infected *C. albicans* ATCC vs. *C. albicans* RVVC. (**C**) Percentage and absolute number of mBD1+ cells in vaginal lavage from unestrogenized, uninfected, and *C. albicans* ATCC- or *C. albicans* RVVC-infected animals at days 2 and 4 pi evaluated by flow cytometry. Mean ± SEM of three independent experiments (*n* = 8 per group). * *p* < 0.05, *** *p* < 0.001 infected vs. uninfected; + *p* < 0.05; ++ *p* < 0.01; +++ *p* < 0.001 infected vs. unestrogenized; # *p* < 0.05, infected *C. albicans* ATCC vs *C. albicans* RVVC. (**D**) Percentage and absolute number of Gr-1+ cells, and (**E**) percentage of Gr-1+/mBD1+ cells in vaginal lavage of different groups at days 2 and 4 pi evaluated by flow cytometry. Mean ± SEM of three independent experiments (*n* = 8 per group). * *p* < 0.05 infected vs. unestrogenized; + *p* < 0.05 infected vs. uninfected; # *p* < 0.05 infected *C. albicans* ATCC vs. *C. albicans* RVVC. ANOVA Test.

To evaluate whether the pathogenicity of the fungal strain can modulate mBD1 expression, mice were infected with a *C. albicans* strain isolated from an RVVC patient or with *C. albicans* ATCC-36801 strain, and mBD1 expression was evaluated. When their virulence profiles were compared, *C. albicans* RVVC proved to be more virulent than the collection strain. The fungal burden (D2 pi) (Figure 2A) and IL-1β amounts (Figure 2B) were higher when the mice were inoculated with the clinical isolate. Moreover, a significant increase in the percentage of mBD1+ cells was observed in VL from animals infected with *C. albicans* RVVC when compared with the other groups (D2 pi) (Figure 2C). At D4 pi, the percentage and the absolute number of mBD1+ cells were significantly increased compared to both negative controls. The results showed a distinct profile of AMP induction depending on the strain origin, with an exacerbated mBD1+ expression when the infection was induced with the more virulent strain.

The PMN recruited into the vaginal lumen during AVVC contributed to tissue damage and the local inflammation associated with vaginitis symptoms. The infection generated by the clinical strain did not produce variations in the percentage and absolute number of PMNs (Gr-1+ cells) recruited to the vaginal lumen when compared with the animals infected with *C. albicans* ATCC-36801 (Figure 2D, left and right panels). However, the percentage of Gr-1+ mBD1+ cells was significantly higher (D2 pi) in mice infected with *C. albicans* RVVC strain than in the other experimental groups (Figure 2E). A similar result was observed when the absolute number of Gr-1+ mBD1+ cells were compared. These data indicate that the number of PMN recruited to the vaginal lumen was independent of the *C. albicans* strain used. Nevertheless, the percentage of mBD1-producing PMNs was higher when the infection was induced with a clinical strain.

### 3.3. Intravaginal Cytokines Profile and Virulence Factors of Clinical Isolates from Patients with AVVC or RVVC

To understand the role of hBD in patients with AVVC or RVVC, we evaluated clinical-related parameters, characteristics of the etiological agent, and local production of cytokines in both groups of patients. We found that the percentage of risk factors was higher in the AVVC than in the RVVC population (Table 1) and that *C. albicans* was the prevalent species in both forms of the disease. *C. albicans* strains recovered from patients showed a similar frequency of SAP-producing isolates, but a higher enzyme activity was detected in the AVVC group. The number of LIP-producing strains was lower among the RVVC isolates than in AVVC, but the enzyme activity was similar in both groups.

**Figure 3:**
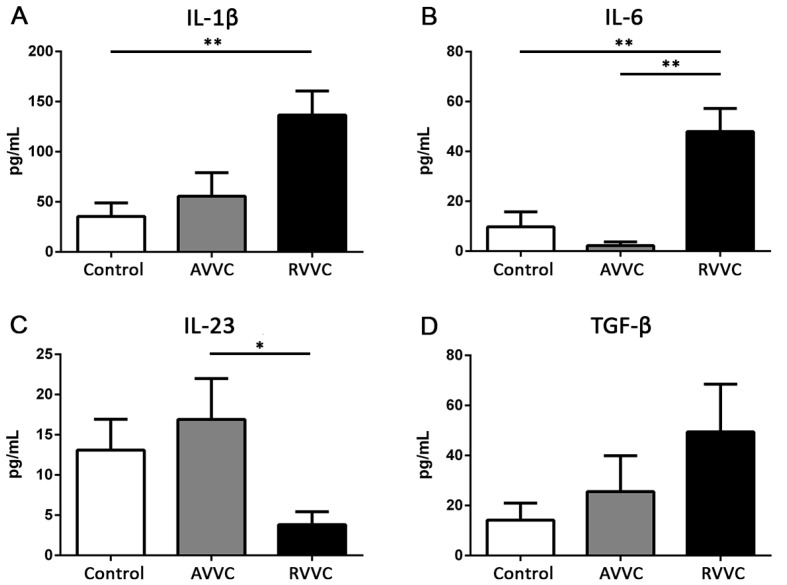
Cytokines concentration in cervicovaginal lavage samples from patients of the Control (white bars), AVVC (grey bars), and RVVC (black bars) groups, quantified by ELISA assay. (**A**) IL-1β (**B**) IL-6, (**C**) IL-23, and (**D**) TGF-β. The assay could not be performed in all the patients of each cohort due to sample limitations. Mean ± SEM. Control *n* = 26, AVVC *n* = 18, RVVC *n* = 48. * *p* < 0.05, ** *p* < 0.01. ANOVA test.

The symptoms of *Candida* vaginitis suggest an active vaginal inflammatory process resulting from an imbalance in the local microenvironment. IL-1β, IL-6, TNF-α, IL-17, IL-22, IL-23, and TGF-β concentrations were determined in CVL from Control, AVVC, and RVVC groups (Figure 3). Patients with RVVC showed higher levels of IL-1β and IL-6 in CVL than Control individuals and higher IL-6 levels than patients with the acute form of the mycosis. TNF-α was undetectable in all the evaluated samples. Moreover, while TGF-β levels showed some variations between the groups, these changes were not statistically significant. Furthermore, IL-23 significantly decreased in CVL from patients with RVVC compared to those with AVVC, and both IL-17 and IL-22 were undetectable in all the groups studied. These results indicate that the cytokine profile of RVVC patients is characterized by high amounts of IL-1β and IL-6, low concentration of IL-23, and undetectable levels of IL-17, IL-22, and TNF-α.

### 3.4. Study of AMPs of the BD Family in Patients with AVVC and RVVC

**Figure 4:**
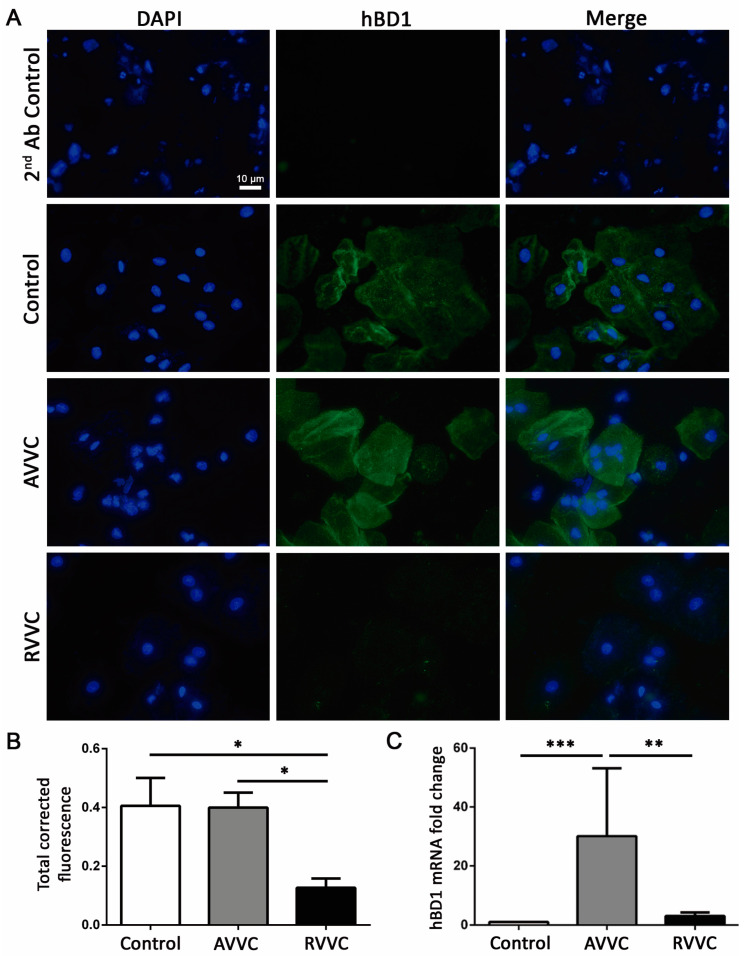
Human Beta Defensin-1 (hBD1) expression in cells of cervicovaginal lavage. (**A**) Immunofluorescence of cervicovaginal lavage cytospin samples from Control individuals and patients with AVVC or RVVC. Upper panel (2nd Ab control), cytospin of cervicovaginal lavage from patients with RVVC. Cell nuclei were stained with DAPI (blue), and hBD1 expression is shown in green. Images are shown at ×630 magnification. (**B**) Fluorescence semiquantification was performed using the ImageJ software. The assay could not be performed in all the patients of each cohort due to sample limitations. Mean ± SEM. Control *n* = 4, AVVC *n* = 6, RVVC *n* = 5. * *p* < 0.05. (**C**) hBD1 mRNA expression by real-time PCR in cervicovaginal lavage cells from Control individuals and patients with AVVC or RVVC. The assay could not be performed in all the patients of each cohort due to sample limitations. Mean ± SEM. Control *n* = 14, AVVC *n* = 5, RVVC *n* = 15. ** *p* < 0.01, *** *p* < 0.001. ANOVA test.

The modulation of mBD1 in the vaginal mucosa in response to *Candida* infection suggests an active role of this AMP during AVVC. To corroborate this hypothesis, we explored hBD transcript and protein expression in CVL cells of the AVVC and RVVC groups. Interestingly, the hBD1 mRNA level was increased in the AVVC group compared to the Control and RVVC groups (Figure 4C). The immunostaining assay showed that cells from healthy individuals exhibited a basal hBD1 expression, while EC from patients with AVVC showed a more homogeneous hBD1 expression in cell cytoplasm (Figure 4A). Further, the expression of hBD1 was significantly decreased in RVVC samples compared with those from healthy individuals and patients with AVVC (Figure 4B).

**Figure 5:**
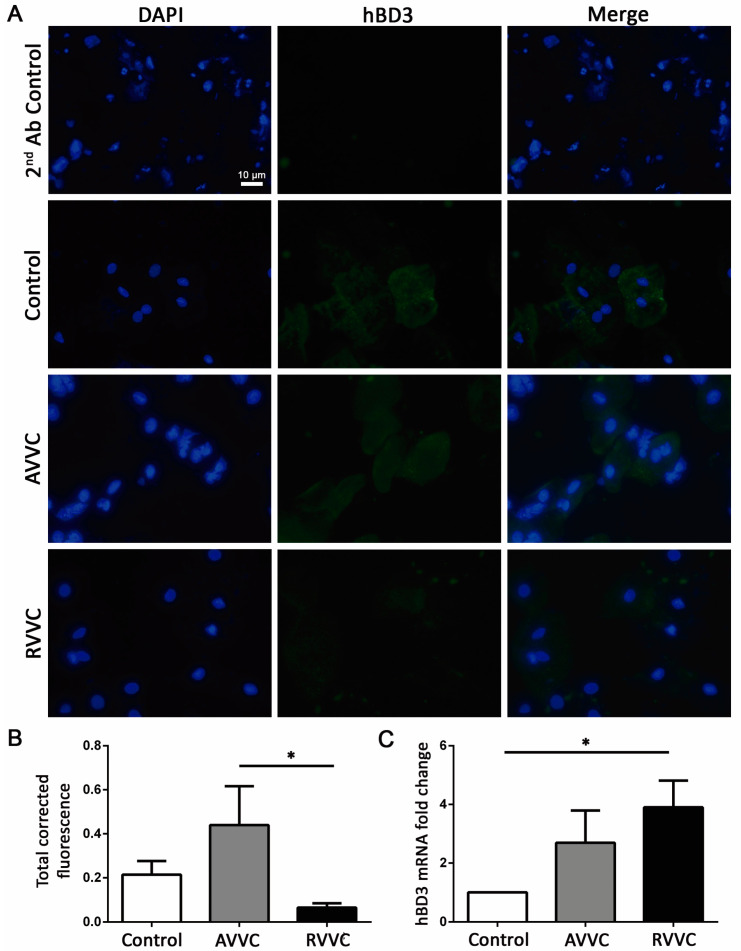
Human Beta Defensin-3 (hBDR3) expression in cells of cervicovaginal lavage. (**A**) Immunofluorescence of cervicovaginal lavage cytospin samples from Control individuals and patients with AVVC or RVVC. Upper panel (2nd Ab control), cytospin of cervicovaginal lavage from patients with RVVC. Cell nuclei were stained with DAPI (blue), and hBD3 expression is shown in green. Images are shown at ×630 magnification. (**B**) Fluorescence semiquantification analyzed with ImageJ software. The assay could not be performed in all the patients of each cohort due to sample limitations. Mean ± SEM. Control *n* = 4, AVVC *n* = 6, RVVC *n* = 5; * *p* < 0.05. (**C**) hBD3 mRNA expression by real-time PCR in cervicovaginal lavage cells from Control individuals and patients with AVVC or RVVC. The assay could not be performed in all the patients of each cohort due to sample limitations. Mean ± SEM. Control *n* = 14, AVVC *n* = 5, RVVC *n* = 15; * *p* < 0.05. ANOVA test.

We also evaluated hBD2 and hBD3 mRNA expression in CVL cells of the different groups. Although the levels of the hBD2 transcript showed no significant changes between the tested patients (data not shown), hBD3 mRNA was upregulated in RVVC samples compared to those of the Control group (Figure 5C). Similar to that observed for constitutive hBD1 expression, hBD3 level in the RVVC group was significantly decreased when compared to the AVVC group (Figure 5A,B).

### 3.5. Regulation of hBD1 in Epithelial Cells of Female Genital Tract

**Figure 6:**
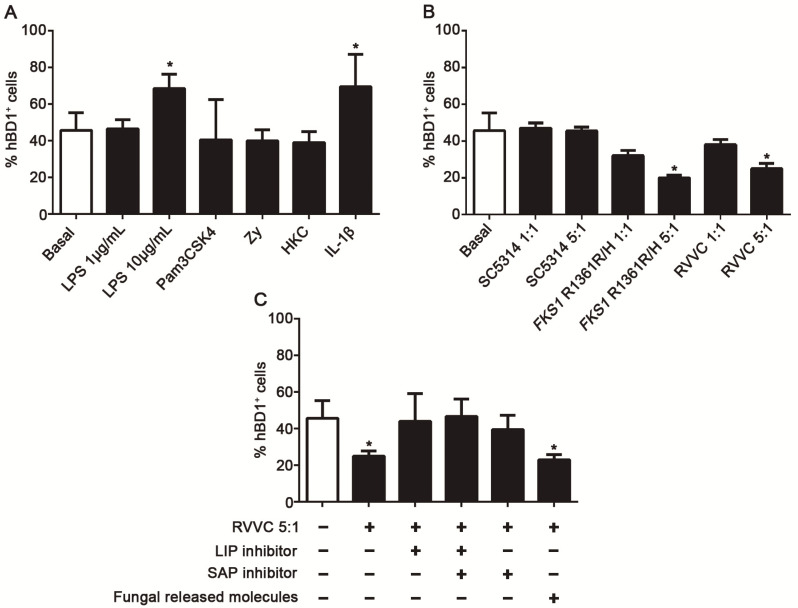
Regulation of human Beta Defensin-1 (hBD1) in epithelial cells of female genital tract. (**A**) Percentage of hBD1 positive HeLa cells incubated for 4 h without stimuli (basal) or with LPS (1 μg/mL or 10 μg/mL), Pam3CK4 100 ng/mL, IL-1β 10 ng/mL, HKC 1:1 ratio, or Zymosan 20 μg/mL, determined by flow cytometry. Data are representative of three independent experiments. Mean ± SEM. * *p* < 0.05 compared to basal expression. (**B**) Percentage of hBD1-positive HeLa cells incubated 4 h without stimuli (basal) or with different *C. albicans* strains (*C. albicans SC5314, C. albicans FKS1 R1361R/H*, and *C. albicans* isolated from an RVVC patient) at 1:1 or 5:1 ratio. Data are representative of three independent experiments. Mean ± SEM.* *p* < 0.05 compared to basal expression. (**C**) Percentage of hBD1-producing cells after 4 h of transwell system culture without stimuli (basal) or with *C. albicans* RVVC at a 5:1 ratio; *C. albicans* RVVC at a 5:1 ratio together with a SAP inhibitor Pepstatin (1 μg/mL) or the LIP inhibitor ASA (625 μM) and with both inhibitors. Data are representative of three independent experiments. Mean ± SEM.* *p* < 0.05 compared to basal. ANOVA test.

In order to elucidate the signals involved in the modulation of hBD1 expression in EC of the female genital tract, we used a HeLa cell line in vitro model [26,30] and determined hBD1+ cells percentage in basal condition and after exposure to different stimuli. Constitutive hBD1 expression was observed in the basal condition, and a significant increase in hBD1+ cells percentage was found after stimulation with 10 ug/mL of LPS (TLR4 agonist) and recombinant hIL-1β (Figure 6A). Nevertheless, Pam3CSK4 (TLR1/2), Zymosan (TLR2 and Dectin-1 agonists), and HKC were not able to modulate hBD1 expression. Further, the ability of different viable *C. albicans* strains to regulate hBD1 expression was evaluated. We found a significant decrease in hBD1 expression in EC cultured with the *C. albicans* clinical isolates FKS1-R1361R/H and RVVC at a 5:1 ratio (Figure 6B); however, no changes were observed after exposure to *C. abicans* SC5314. These results show that fungal strain origin and inoculum size are relevant factors during *C. albicans* interaction with host EC.

The next step was to establish whether direct fungal contact or released soluble factors were involved in the described phenomenon. Thus, HeLa cells were cultured with *C. albicans* RVVC using a transwell system. The results showed that, in the presence of fungal released molecules alone, the percentage of hBD1+ cells significantly decreased compared to the basal condition (Figure 6C). Considering the role of *C*. *albicans* SAP family in vaginitis [31,32], previous results about fungal LIP [26,33], and the enzymatic activity we have detected in clinical isolates (Table 1), HeLa cells were also cultured in the presence of SAP and LIP inhibitors. Interestingly, hBD1 decreased expression was restored to its basal values after exposure to both drugs, supporting the notion that SAP and LIP are involved in the downregulation of hBD1, both together and separately.

## 4. Discussion

BDs are an important alarmin family within the AMPs molecules involved in the protection against pathogens in mucosal tracts [10,12,13]. Despite these properties, studies on their roles during *C. albicans* vaginal infection in animal models and especially in patients with the acute or recurrent form of the mycosis are limited. Using a murine AVVC model, we described the kinetic response of mBD1 expression in the vaginal tract during *C. albicans* ATCC-36801 infection. The number of mBD1+ cells in the lumen at D2 pi was significantly increased, and mBD1+ protein expression was observed in resident cells and recruited PMNs. These results were confirmed in an in vivo infection model using a *C. albicans* strain isolated from RVVC. We found that the fungal load and intravaginal level of IL-1β remained high, mBD1+ cells increased, and the percentage of Gr-1+ mBD1+ cells was nearly 2.8-fold higher than that found in *C. albicans* ATCC-36801-infected animals. These findings showed that, after acute exposition to *C. albicans*, constitutive mBD1 expression was early increased and that this response was correlated with the infection severity. In line with this result, we had previously reported that lack of TLR2 receptor signaling [23], which predisposes mice to massive *C. albicans* invasion of the vaginal tract, leads to an exacerbated expression of mBD1+ and IL-1β, indicating that there is a strong correlation between both mediators and the immunopathological response during VVC.

BDs production depends on the cellular type, stimulus, environment, and context of the cells being stimulated [10,34]. Pahl et al. [16] have reported, in an OE21 esophageal cell model, that the blockade of IL-1β receptor-dependent signaling strongly reduces the induction of hBD, and they also describe a signaling loop of IL-1β and hBD regulation. Several in vitro results have confirmed this report [35,36,37]. The IL-1β canonical pathway is regulated at the transcriptional level through fungal PAMPs recognition and protein secretion by the NLRP3 inflammasome [38]. The role of IL-1β during VVC has been well-documented in animal models [7,23,32,39], in works with Nlrp3^−/^^−^ mice [40], and in patients with NLRP3 polymorphisms [2,41]. In addition, the SAP family members are involved in IL-1β induction [31,32]. Our findings on murine AVVC and the effect of recombinant hIL-1β on hBD1 induction in in vitro cultures of epithelial cells of the genital tract agree with the available evidence about IL-1β and hBD1 induction in the vaginal tract.

Although it is accepted that the mucosal microenvironment regulates BDs expression, only a few studies have explored cytokines secretion during *C. albicans* infection in the female genital tract [31,41,42,43]. Unfortunately, the groups of patients included in those studies are not comparable between them, nor with those of our study. We evaluated the concentration of different cytokines in CVL of patients with AVVC and RVVC and healthy individuals and observed that the CVL cytokine profile of women with RVVC was characterized by high levels of IL-1β and IL-6, low concentration of IL-23, and undetectable levels of TNF-β, IL-17, and IL-22. Regarding IL-23, Ryan et al. [44] have proposed that, during Chronic Mucocutaneous Candidiasis, low levels of this cytokine produce deregulation of the IL-6/IL-23/IL-17 axis and impact the ability to mount and sustain an efficient antifungal response. In our study, we observed low local production of IL-17 (<15 pg/mL) in both the acute and recurrent form of this mycosis, confirming that this cytokine is dispensable for the immunopathogenesis of VVC. These results agree with the notion that humans with inborn errors related to IL-17 immunity do not seem to be more susceptible to *Candida* vaginal infections [45,46,47]. Recently, Peter et al. [5] have demonstrated, in models of estrogen-induced VVC, that mice lacking IL-17RA, Act1, and IL-22 do not show increased susceptibility to *Candida* vaginitis. Taking together, these results highlight our understanding of the controversial role of Th17/IL-17 axis signaling in the immunopathogenesis of VVC [5,23,39,48].

Considering the inflammatory milieus observed during RVVC (higher IL-1β and IL-6), a novel and unsuspected result was the hBD1 expression observed in EC from patients with RVVC. Even though in the acute form of the infection hBD1 mRNA level was increased and its cytoplasmic expression presented homogeneous distribution, during RVVC, both hBD1 mRNA and protein expressions were severely downmodulated. Furthermore, hBD3 mRNA expression was upregulated, whereas its protein expression was significantly reduced, suggesting a phenomenon of posttranscriptional inhibition. These results demonstrated a significant reduction of constitutive and inducible members of the BD family in patients with RVVC. Host and fungal factors are involved in the outcome of vaginitis [48]. In agreement with our previous results [26,33] and those reported by other authors [32,33], we found that a high proportion of AVVC and RVVC clinical isolates showed active SAP and LIP secretion. Our in vitro VVC model approach provides data on the stimuli able to trigger hBD1 induction but also provides information on pathogen-EC interactions. The data linking strain origin and fungal burden suggest the existence of a critical threshold at which clinical strains can inhibit hBD1 expression. The use of enzyme inhibitors allowed us to determine that SAP and LIP were involved in this phenomenon. Considering the critical role of BDs on the mucosal surfaces, their decrease would constitute a highly efficient escape strategy for *C. albicans*. This finding could explain, at least in part, the low levels of both hBD1 and hBD3 in patients with RVVC, in a context of recurrent fungal overgrowth, invasion, active release of virulence factors [1,32,33,49], and high levels of inflammatory mediators, such as IL-1β, which in another context would stimulate BDs production. This fact and the deregulation of the IL-6/IL-23/IL-17 axis would contribute to *C. albicans* survival and persistence.

It has been shown that certain virulence factors released by pathogens are involved in the downregulation of hBD production; however, most of the existing evidence derives from studies on bacterial infections. In this respect, Patel et al. [37] found that gastric biopsies from patients infected with *Helicobacter pylori* have significantly decreased levels of hDB1 transcripts. Working with gastric epithelium cell lines and different bacteria strains, they proved that this effect depends on the pathogenicity of the strain and is mediated by *H. pylori* virulence factor CagA [29,35]. Chakraborty et al. [50] evaluated the modulation of hBD1 in human EC of the intestinal mucosa exposed to *Vibrio cholerae* and enterotoxigenic *Escherichia coli*. In both cases, essential virulence factors (choleric toxin and labile toxin) were responsible for hBD1 transcription inhibition. This phenomenon has also been described in virus [34]. The in vitro data obtained in our study demonstrate that fungal virulence factors can inhibit hBD1 protein expression in epithelial cells of the genital tract. Thus, our results and extend this conserved evolutionary strategy to fungus–host interaction.

This work identifies a novel role for mBD1 and hBD1 in host fungal defense in the female genital tract and in the pathogenesis of VVC. The knowledge provided on the mechanisms governing the interactions between *C. albicans* and its host is relevant for the proposal and development of new or combined treatment strategies against this mycosis.

## Figures and Tables

**Table 1:** Some characteristics of patients and virulence factors of clinical isolates.

Disease Patients	Age Mean ± SEM	Risk Factor	*C. albicans* Prevalence	Virulence Factors over 20 Clinical Isolates					
				SAP			LIP		
				Frequency (%)	*Pz* Range	*Pz* Mean ± SEM	Frequency (%)	*Pz* Range	*Pz* Mean ± SEM
AVVC (*n* = 20)	27.8 ± 6.7	73 %	93%	12/20 (60%)	1.00–2.57	1.62 ± 0.61 *	12/20 (60%)	1.00–4.00	1.72 ± 0.84
RVVC (*n* = 59)	35.2 ± 7.6	43 %	96%	14/20 (70%)	1.00–1.70	1.32 ± 0.28	8/20 (40%)	1.00–2.66	1.46 ± 0.68

Risk factors evaluated: oral contraceptives, antibiotic treatment, or a combination of both. They were calculated over the total number of patients in each group. SAP: acid aspartic protease; LIP: lipase; * *p* < 0.05 *t* test.

## Supplementary Materials

The following are available online at https://www.mdpi.com/article/10.3390/jof8010020/s1, Figure S1: Dot plot shows the gate strategy as follows: to exclude dead cell and aggregates from analysis, cells were first gated on excluding the smallest cells and debris region and then by gating on singlets regions based on FSC-A vs. FSC-H and SSC-A vs. SSC-H. Then Gr-1 + cells and BD-1+ cells were determined.
