# Supplementary material for: Candida albicans Modulates Murine and Human Beta Defensin-1 during Vaginitis
Source: J Fungi (Basel). 2021 Dec 28;8(1):20. doi: 10.3390/jof8010020 (PMC8778459; doi:10.3390/jof8010020)
Supplement: Supplementary file 1 [file jof-08-00020-s001.zip › jof-1450666-supplementary.pdf]

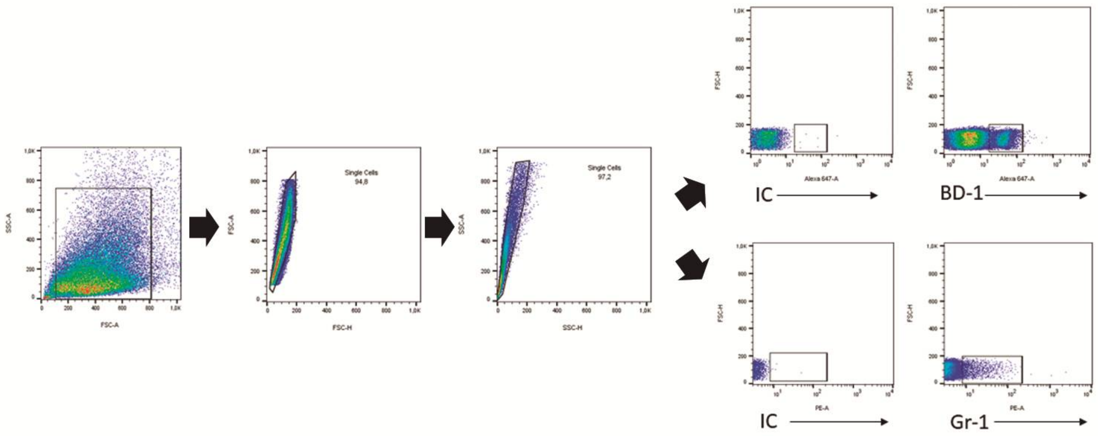

Figure S1. Dot plot shows the gate strategy as follow: to exclude dead cell and aggregates from analysis, cells were first gated on excluding the smallest cells and debris region and then by gatting on singlets regions based on FSC-A vs. FSC-H and SSC-A vs. SSC-H. Then Gr-1 + cells and BD-1+ cells were determined.
